# Supplementary material for: Quantitative Proteogenomic Characterization of Inflamed Murine Colon Tissue Using an Integrated Discovery, Verification, and Validation Proteogenomic Workflow
Source: Proteomes. 2022 Apr 14;10(2):11. doi: 10.3390/proteomes10020011 (PMC9036229; doi:10.3390/proteomes10020011)

Supporting information for "Quantitative Proteogenomic Characterization of Inflamed Murine Colon Tissue Using an Integrated Discovery, Verification, and Validation Proteogenomic Workflow"

Andrew T. Rajczewski [1], Qiyuan Han [1], Subina Mehta [1], Praveen Kumar [1], Pratik D. Jagtap [1], Charles G. Knutson [2], James G. Fox [2], Natalia Y. Tretyakova [3] and Timothy J. Griffin [1,*]

[1] Department of Biochemistry, Molecular Biology and Biophysics, University of Minnesota, Minneapolis, MN 55455, USA; rajcz001@umn.edu (A.T.R.); hanxx963@umn.edu (Q.H.); smehta@umn.edu (S.M.); pravs3683@gmail.com (P.K.); pjagtap@umn.edu (P.D.J.)

[2] Department of Biological Engineering, Massachusetts Institute of Technology, Cambridge, MA 02139, USA; charlie.knutson@novartis.com (C.G.K.); jgfox@mit.edu (J.G.F.)

[3] Department of Medicinal Chemistry, the Masonic Cancer Center, University of Minnesota, Minneapolis, MN 55455, USA; trety001@umn.edu

* Correspondence: tgriffin@umn.edu

Table of Contents

Table S1: Proximal colon samples....................S-2

Table S2: Inclusion list for targeted detection of non-canonical peptides....................S-3

Table S3: Non-canonical peptide sequences....................S-7

Table S4: Human parallels of mouse non-canonical peptides....................S-9

Figure S1: Genomic coordinated of protein enriched in inflamed colon samples....................S-10

Figure S2: MS/MS spectra of non-canonical peptides passing PepQuery validation S-11

Figure S3: Genomic coordinates of non-canonical peptide AASSANIPK S-14

Figure S4: Genomic coordinates of non-canonical peptide AASSANIPK....................S-15

Table S1: Proximal colon samples. Proximal colon samples used in this study, with the sample accession numbers, animal identification numbers, nature of the tissue, and the tandem mass tag mass assigned in LC-MS experiments. Accession numbers and animal identification numbers were assigned by the Tannenbaum lab, where the samples were first generated and harvested for future analysis.

**Accession #**	**Mouse ID**	**Sample Type**	**TMT-6plex label**
12-5632	5812	Control	126
12-5633	5813	Control	127
12-5634	5833	Control	128
12-5646	5874	H hepaticus infected	129
12-5647	5819	H hepaticus infected	130
12-5648	5820	H hepaticus infected	131

Table S2: Inclusion list for targeted detection of non-canonical peptides in proximal colon samples. Based on the initial global proteomics data, m/z values and charge states were determined for putative non-canonical peptides and used to create this inclusion list for targeted PRM analyses.

Mass [m/z]	CS [z]	Polarity	Detection Start [min]	Detection End [min]	(N)CE	(N)CE type	Comment
401.91379	3	Positive	31.59	46.59	35	NCE	aSLQVSTLR
411.58926	3	Positive	38.71	53.71	35	NCE	dSILQAk
417.92569	3	Positive	48.28	63.28	35	NCE	aEPGLPLGLR
423.2294	3	Positive	49	64	35	NCE	dPsAIGk
436.7355	4	Positive	36.75	51.75	35	NCE	ssVRIGSGSWk
446.49326	4	Positive	42.84	57.84	35	NCE	iLGAILAMASTQsR
446.49326	4	Positive	42.84	57.84	35	NCE	iLGAILAMAStQSR
448.62711	3	Positive	52.49	67.49	35	NCE	lLGIDLGGk
466.93878	3	Positive	37.24	52.24	35	NCE	aPPTWPGSk
472.79034	2	Positive	34.13	49.13	35	NCE	lSANLR
472.79095	2	Positive	37.12	52.12	35	NCE	lSANLR
480.94556	3	Positive	37.94	52.94	35	NCE	aPPTWPGSk
484.29071	3	Positive	60.79	75.79	35	NCE	ePILtVLk
484.29514	3	Positive	51.78	66.78	35	NCE	ePILtVLk
491.6076	3	Positive	57.64	72.64	35	NCE	qVIyELk
491.60995	3	Positive	57.68	72.68	35	NCE	qVIyELk
491.61108	3	Positive	58.19	73.19	35	NCE	qVIyELk
491.97314	3	Positive	50.25	65.25	35	NCE	vMPILLDSk
498.2988	3	Positive	59.58	74.58	35	NCE	ePILtVLk
501.95837	3	Positive	49.67	64.67	35	NCE	eEEGLEVLk
502.27893	3	Positive	36	51	35	NCE	iTEHsIPk
507.60172	3	Positive	57.31	72.31	35	NCE	aPPtWPGSk
507.60608	3	Positive	43.38	58.38	35	NCE	aPPTWPGsk
507.96249	3	Positive	51.86	66.86	35	NCE	qMINLTESk
508.80139	2	Positive	39.06	54.06	35	NCE	iTNLER
508.80185	2	Positive	37.78	52.78	35	NCE	iTNLER
512.30438	3	Positive	53.21	68.21	35	NCE	eVmLVGIGDk
515.62384	3	Positive	50.35	65.35	35	NCE	dLSLEGPEGk
520.30646	3	Positive	40.46	55.46	35	NCE	qHFPSMILk
520.31342	3	Positive	39.32	54.32	35	NCE	qHFPSMILk
525.6405	3	Positive	59.44	74.44	35	NCE	qHFPSmILk
532.32422	2	Positive	34.02	49.02	35	NCE	tSSISALR
532.32428	2	Positive	36.54	51.54	35	NCE	tSSISALR
534.06195	4	Positive	64.43	79.43	35	NCE	skPcISGLMVPEk
538.32654	2	Positive	2.83	17.83	35	NCE	iFSLNPR
538.68396	3	Positive	62.27	77.27	35	NCE	lSANLRLLQk
547.97961	3	Positive	49.92	64.92	35	NCE	iFsLNPRSk
553.32477	2	Positive	37.54	52.54	35	NCE	tSSISALR
554.05615	4	Positive	61.25	76.25	35	NCE	skPcIsGLMVPEk
554.05646	4	Positive	60.61	75.61	35	NCE	skPcIsGLMVPEk
556.00024	3	Positive	53.53	68.53	35	NCE	skPAItGPk
559.3717	2	Positive	40.09	55.09	35	NCE	iLQLVFR
559.37177	2	Positive	39.57	54.57	35	NCE	iLQLVFR
561.2959	3	Positive	66.82	81.82	35	NCE	wTSEFEASLINR
561.29816	3	Positive	67.18	82.18	35	NCE	wTSEFEASLINR
561.2984	3	Positive	67.61	82.61	35	NCE	wTSEFEASLINR
561.29858	3	Positive	67.35	82.35	35	NCE	wTSEFEASLINR
561.29871	3	Positive	67.09	82.09	35	NCE	wTSEFEASLINR
564.30493	3	Positive	53.33	68.33	35	NCE	dIELVmAQANVSR
564.82117	4	Positive	56.45	71.45	35	NCE	pIRPGHYPASSPTAVHAIR
566.3244	3	Positive	49.81	64.81	35	NCE	fSmVVQDGIVk
566.32886	3	Positive	54.31	69.31	35	NCE	fSmVVQDGIVk

572.29749	2	Positive	45.95	60.95	35	NCE	tsSISALR
572.29749	2	Positive	45.95	60.95	35	NCE	tSsISALR
572.29749	2	Positive	45.95	60.95	35	NCE	tSSISALR
573.35065	2	Positive	33.77	48.77	35	NCE	dPSAIGk
573.35089	2	Positive	36.48	51.48	35	NCE	dPSAIGk
573.35095	2	Positive	36.3	51.3	35	NCE	dPSAIGk
573.35175	2	Positive	35.75	50.75	35	NCE	dPSAIGk
574.89465	2	Positive	55.83	70.83	35	NCE	fIVAIk
574.89545	2	Positive	58.63	73.63	35	NCE	fIVAIk
574.99872	3	Positive	42.6	57.6	35	NCE	fSMVVQDGIVk
580.97656	3	Positive	43	58	35	NCE	iLGAILAMAsTQSR
580.97656	3	Positive	43	58	35	NCE	iLGAILAMAStQSR
582.38208	2	Positive	49.62	64.62	35	NCE	aGAVFLk
582.38281	2	Positive	46.6	61.6	35	NCE	aGAVFLk
582.65967	3	Positive	39.77	54.77	35	NCE	skPAItGPk
582.85614	2	Positive	45.1	60.1	35	NCE	fPDTVk
586.31213	3	Positive	50.98	65.98	35	NCE	iLGAILAmAStQSR
586.31213	3	Positive	50.98	65.98	35	NCE	iLGAILAmAsTQSR
586.31213	3	Positive	50.98	65.98	35	NCE	iLGAILAmASTQsR
586.31317	3	Positive	52.02	67.02	35	NCE	iLGAILAmAStQSR
586.31317	3	Positive	52.02	67.02	35	NCE	iLGAILAmAsTQSR
586.3418	2	Positive	10.23	25.23	35	NCE	aNNINIQR
592.32434	2	Positive	46.47	61.47	35	NCE	nPTsVk
592.32434	2	Positive	46.47	61.47	35	NCE	nPtSVk
594.99884	3	Positive	57.77	72.77	35	NCE	iQStNQILEAk
594.99884	3	Positive	57.77	72.77	35	NCE	iQsTNQILEAk
599.3103	2	Positive	46.03	61.03	35	NCE	iFsLNPR
603.38831	2	Positive	48.39	63.39	35	NCE	aGAVFLk
605.5661	4	Positive	55.14	70.14	35	NCE	nPTSVkYVEMsSVFHR
606.992	3	Positive	65.75	80.75	35	NCE	fsmVVQDGIVk
607.35724	3	Positive	37.86	52.86	35	NCE	aGPGRPAAAGGAAVRRR
609.56299	4	Positive	55.21	70.21	35	NCE	nPtSVkYVEmSSVFHR
609.56311	4	Positive	51.65	66.65	35	NCE	nPtSVkYVEmSSVFHR
613.33069	2	Positive	56.48	71.48	35	NCE	dPsAIGk
613.33521	2	Positive	49.48	64.48	35	NCE	dPsAIGk
613.33521	2	Positive	49.48	64.48	35	NCE	nPtSVk
613.33521	2	Positive	49.48	64.48	35	NCE	nPTsVk
613.33813	2	Positive	48.97	63.97	35	NCE	dPsAIGk
616.85022	2	Positive	66.38	81.38	35	NCE	sAPLLLGPR
616.88525	2	Positive	37.28	52.28	35	NCE	dSILQAk
616.88544	2	Positive	40.03	55.03	35	NCE	dSILQAk
634.33527	2	Positive	5.67	20.67	35	NCE	dPsAIGk
634.33765	2	Positive	39.9	54.9	35	NCE	dPsAIGk
637.88483	2	Positive	43.44	58.44	35	NCE	dSILQAk
637.88605	2	Positive	44.69	59.69	35	NCE	dSILQAk
637.89258	2	Positive	43.04	58.04	35	NCE	dSILQAk
638.3465	2	Positive	38.01	53.01	35	NCE	hQsALVRR
638.34955	2	Positive	49.25	64.25	35	NCE	hQsALVRR
638.34967	2	Positive	43.96	58.96	35	NCE	hQsALVRR
638.35046	3	Positive	63.8	78.8	35	NCE	iLGAILAmAStQSRR
638.35046	3	Positive	63.8	78.8	35	NCE	iLGAILAmAsTQSRR
638.35046	3	Positive	63.8	78.8	35	NCE	iLGAILAmASTQsRR
652.71063	3	Positive	64.41	79.41	35	NCE	vVLLGLsSIPSLVGHR
652.71063	3	Positive	64.41	79.41	35	NCE	vVLLGLSsIPSLVGHR
652.71124	3	Positive	64.06	79.06	35	NCE	vVLLGLSsIPSLVGHR
652.71124	3	Positive	64.06	79.06	35	NCE	vVLLGLsSIPSLVGHR
658.896	2	Positive	41.94	56.94	35	NCE	aASSANIPk
658.89929	2	Positive	51.87	66.87	35	NCE	aASSANIPk
658.90131	2	Positive	36.53	51.53	35	NCE	aASSANIPk
672.43634	2	Positive	51.71	66.71	35	NCE	lLGIDLGGk

672.4375	2	Positive	54.69	69.69	35	NCE	lLGIDLGGk
672.43762	2	Positive	51.01	66.01	35	NCE	lLGIDLGGk
672.43805	2	Positive	52.45	67.45	35	NCE	lLGIDLGGk
675.922	2	Positive	64.87	79.87	35	NCE	qVIYELk
679.69513	3	Positive	36.75	51.75	35	NCE	aRPVSSAASVYAGAGGSGSR
685.95459	2	Positive	57.54	72.54	35	NCE	ePILTVLk
685.96118	2	Positive	50.13	65.13	35	NCE	ePILTVLk
697.37695	3	Positive	56.46	71.46	35	NCE	aAAAAAAAAAAAASHsVAk
697.37695	3	Positive	56.46	71.46	35	NCE	aAAAAAAAAAAAAsHSVAk
699.9046	2	Positive	37.32	52.32	35	NCE	aPPTWPGSk
699.91199	2	Positive	45.54	60.54	35	NCE	aPPTWPGSk
699.91302	2	Positive	50.85	65.85	35	NCE	aPPTWPGSk
701.12836	4	Positive	55.4	70.4	35	NCE	vLNGPEGDGVPEAVVtLNNQIk
704.37402	2	Positive	46.97	61.97	35	NCE	sLAALPEELR
716.38727	2	Positive	34.14	49.14	35	NCE	yANNNSkY
716.39734	2	Positive	48.99	63.99	35	NCE	rHQsALVRR
719.88727	2	Positive	47.45	62.45	35	NCE	aASsANIPk
719.88727	2	Positive	47.45	62.45	35	NCE	aAsSANIPk
720.35889	3	Positive	42.24	57.24	35	NCE	aRPVSsAASVYAGAGGSGSR
720.35889	3	Positive	42.24	57.24	35	NCE	aRPVsSAASVYAGAGGSGSR
725.9325	2	Positive	60.62	75.62	35	NCE	ePILtVLk
725.93677	2	Positive	51.71	66.71	35	NCE	ePILtVLk
726.39618	2	Positive	41.09	56.09	35	NCE	aMADELSEk
731.90918	2	Positive	49.45	64.45	35	NCE	iTEHsIPk
734.38593	2	Positive	61.01	76.01	35	NCE	amADELSEk
736.90778	2	Positive	53.64	68.64	35	NCE	qVIyELk
736.91187	2	Positive	58.47	73.47	35	NCE	qVIyELk
736.91193	2	Positive	58.13	73.13	35	NCE	qVIyELk
736.9649	2	Positive	41.2	56.2	35	NCE	eLkEVIQR
737.39844	2	Positive	37.31	52.31	35	NCE	rHQsALVRR
737.45685	2	Positive	50.15	65.15	35	NCE	vMPILLDSk
745.45398	2	Positive	49.12	64.12	35	NCE	vmPILLDSk
747.37585	4	Positive	57.2	72.2	35	NCE	tGDFQLHTNVNDGTEFGGSIYQk
747.38037	4	Positive	71.19	86.19	35	NCE	tGDFQLHTNVNDGTEFGGSIYQk
751.92773	2	Positive	51.69	66.69	35	NCE	dLSLEGPEGk
752.91663	2	Positive	47.04	62.04	35	NCE	itEHSIPk
759.95209	2	Positive	50.49	65.49	35	NCE	eVMLVGIGDk
767.38489	4	Positive	73.23	88.23	35	NCE	lcYVALDFEQEMAMVASSSSLEk
779.40741	3	Positive	64.94	79.94	35	NCE	pIRPGHyPASSPTAVHAIR
787.37225	4	Positive	74.06	89.06	35	NCE	lcyVALDFEQEMAMVASSSSLEk
787.37384	4	Positive	72.95	87.95	35	NCE	lcYVALDFEQEMAMVAsSSSLEk
787.37384	4	Positive	72.95	87.95	35	NCE	lcYVALDFEQEMAMVASsSSLEk
787.37384	4	Positive	72.95	87.95	35	NCE	lcyVALDFEQEMAMVASSSSLEk
787.37543	4	Positive	73.48	88.48	35	NCE	lcyVALDFEQEMAMVASSSSLEk
791.37518	4	Positive	70.55	85.55	35	NCE	lcyVALDFEQEmAMVASSSSLEk
791.37518	4	Positive	70.55	85.55	35	NCE	lcyVALDFEQEMAmVASSSSLEk
791.37524	4	Positive	70.53	85.53	35	NCE	lcYVALDFEQEMAmVASsSSLEk
791.3764	4	Positive	70.67	85.67	35	NCE	lcyVALDFEQEmAMVASSSSLEk
791.3764	4	Positive	70.67	85.67	35	NCE	lcyVALDFEQEMAmVASSSSLEk
791.37866	4	Positive	73.49	88.49	35	NCE	lcyVALDFEQEmAMVASSSSLEk
791.37866	4	Positive	73.49	88.49	35	NCE	lcyVALDFEQEMAmVASSSSLEk
807.52252	2	Positive	62.4	77.4	35	NCE	lSANLRLLQk
811.48035	2	Positive	37.71	52.71	35	NCE	sSVRIGSGSWk
820.06818	3	Positive	55.43	70.43	35	NCE	pIRPGHYPASsPtAVHAIR
821.80524	3	Positive	84.38	99.38	35	NCE	vHAELADVLTEVVVDSVLAVR
821.80615	3	Positive	85.05	100.05	35	NCE	vHAELADVLTEVVVDSVLAVR
821.80652	3	Positive	84.89	99.89	35	NCE	vHAELADVLTEVVVDSVLAVR
833.50031	2	Positive	53.53	68.53	35	NCE	skPAItGPk
833.50031	2	Positive	53.53	68.53	35	NCE	skPAITGPk
840.99542	2	Positive	58.94	73.94	35	NCE	fSMVVQDGIVk

841.44312	2	Positive	66.86	81.86	35	NCE	wTSEFEASLINR
841.44318	2	Positive	67.64	82.64	35	NCE	wTSEFEASLINR
841.44366	2	Positive	67.1	82.1	35	NCE	wTSEFEASLINR
841.44452	2	Positive	67.39	82.39	35	NCE	wTSEFEASLINR
845.95221	2	Positive	53.45	68.45	35	NCE	dIELVmAQANVSR
848.9881	2	Positive	54.24	69.24	35	NCE	fSmVVQDGIVk
852.01007	2	Positive	46.95	61.95	35	NCE	iQSTNQILEAk
856.9527	2	Positive	49.7	64.7	35	NCE	aSLQVStLRLcR
856.9527	2	Positive	49.7	64.7	35	NCE	aSLQVsTLRLcR
870.96143	2	Positive	43.01	58.01	35	NCE	iLGAILAMAsTQSR
873.51385	2	Positive	50.85	65.85	35	NCE	lAHLILsLEAk
921.84399	3	Positive	62.36	77.36	35	NCE	vLNGPEGDGVPEAVVTLNNQIk
922.46405	3	Positive	68.16	83.16	35	NCE	yVALDFEQEMAMAASSSSLEk
927.7923	3	Positive	71.23	86.23	35	NCE	yVALDFEQEMAmAASSSSLEk
927.7923	3	Positive	71.23	86.23	35	NCE	yVALDFEQEmAMAASSSSLEk
933.12366	3	Positive	67.17	82.17	35	NCE	yVALDFEQEmAmAASSSSLEk
934.50049	3	Positive	55.09	70.09	35	NCE	vLNGPEGDGVPEAVVtLNNQIk
941.79236	3	Positive	70.87	85.87	35	NCE	yVALDFEQEMAmAASSSSLEk
941.79236	3	Positive	70.87	85.87	35	NCE	yVALDFEQEmAMAASSSSLEk
999.16479	3	Positive	81.56	96.56	35	NCE	cYVALDFEQEMAMVASSSSLEk
1022.84576	3	Positive	73.46	88.46	35	NCE	lcYVALDFEQEMAMVASSSSLEk
1036.85205	3	Positive	73.56	88.56	35	NCE	lcYVALDFEQEMAMVASSSSLEk
1049.49646	3	Positive	73.28	88.28	35	NCE	lcyVALDFEQEMAMVASSSSLEk
1049.49756	3	Positive	73.48	88.48	35	NCE	lcyVALDFEQEMAMVASSSSLEk
1049.4989	3	Positive	74.82	89.82	35	NCE	lcYVALDFEQEMAMVAsSSSLEk
1049.505	3	Positive	75.33	90.33	35	NCE	lcyVALDFEQEMAMVASSSSLEk
1054.83679	3	Positive	76.04	91.04	35	NCE	lcYVALDFEQEMAmVAsSSSLEk
1060.17273	3	Positive	74.55	89.55	35	NCE	lcYVALDFEQEmAmVAsSSSLEk
1060.17273	3	Positive	74.55	89.55	35	NCE	lcyVALDFEQEmAmVASSSSLEk
1063.49658	3	Positive	62.88	77.88	35	NCE	lcYVALDFEQEMAMVASSSsLEk
1068.83069	3	Positive	58.57	73.57	35	NCE	lcYVALDFEQEMAmVASSSsLEk
1108.03918	2	Positive	57.93	72.93	35	NCE	ntPQLADIVATGFSVcGR

Table S3: Non-canonical peptide sequences. Non-canonical sequence peptides identified, validated, and quantified in inflamed proximal colon tissues. Peptide precursors with at least three product ions (b- and/or y-ions) were detected in Skyline. A weighted contrast angle of the MS/MS spectra peaks against those of the reference library is reported in Skyline as the dot product, with a score of 1.0 representing a perfect match and 0.0 representing no match

Peptide	Gene/Genetic Coordinates	Phosphorylated	Detected in Targeted	Skyline Dot Product
AAAAAAAAAAAAASHSVAK	Slc4a4		X	0.65
AASSANIPK	Sorl1		X	0.47
AEPGLPLGLR	Sec1		X	0.86
AGAVFLK	chr7:30972023-30972044			
AGPGRPAAAGGAAVRRR	Clstn1			
AMADELSEK	Nucb2			
APPTWPGSK	Slc1a5		X	0.41
ARPVSSAASVYAGAGGSGSR	Akap6		X	0.47
ASLQVSTLRLCR	Zfp219	x	X	0.71
CYVALDFEQEMAMVASSSSLEK	chr2:130657397-130657463			
DIELVMAQANVSR	chr7:45080696-45080735		X	0.78
DIRQMINLTESK	Morc3			
DLSLEGPEGK	Cenpv		X	0.95
DPSAIGK	chr4:109689395-109689416		X	0.61
DSILQAKL	chrX:5699255-5699279		X	0.78
EEEGLEVLK	Sft2d1		X	0.77
ELKEVIQR	chr9:107846694-107846718			
EPILTVLK	Lamb2		X	0.79
EVMLVGIGDK	Ppil4		X	0.56
FIVAIKG	Cpeb2			
FSMVVQDGIVK	chr11:64321586-64321619		X	0.61
GHARSSRMNAFPL	chr9:63755704-63755743			
GIKPVTLELGGK	Rcsd1		X	0.64
IFSLNPRSK	Cipc	x	X	0.79
ILGAILAMASTQSRR	chr12:55637554-55637599		X	0.78
IQSTNQILEAK	chr9:90120273-90120306		X	0.76
ITEHSIPK	Nol10		X	0.81
ITNLERGRER	chr17:66549926-66549956		X	0
KANNINIQRR	Scaf8			
KILQLVFR	chr16:46443630-46443654			
LAHLILSLEAK	chr15_KI270905v1_alt:3773278-31485159	x	X	0.43
LCYVALDFEQEMAMVASSSSLEK	chr2:130657394-130657463	x		
LGTGAMLPLEAVK	chr4:156226782-156226821		X	0.86
LLGIDLGGK	Huwe1		X	0.78
LLYAVNTHCHADHITGSGLLR	chr19:43526299-43526365		X	0.85
LPHLPSILEGRLK	Adprh12			
LQATLQLPQRR	Pm20d1			
LSANLRLLQK	chr8:119530648-119530678	x	X	0.87
NPTSVKYVEMSSVFHR	Zfp729b		X	0.58
NTPQLADIVATGFSVCGRISIIRFPDTVK	Gnb1l	x		
PIRPGHYPASSPTAVHAIR	chr2:149011574-149011631	x	X	0.79
QHFPSMILK	chr3:51956696-51956723		X	0.82
QVIYELK	Elmo1		X	0.75
RHQSALVRR	Cdca4			
SAPLLLGPR	Cyp2s1		X	0.67
SFISLDRVTPR	chr4:16146372-16146405			
SKPAITGPK	Fendrr		X	0.76
SKPCISGLMVPEK	Glu1		X	0.62
SLAALPEELR	Fam214a		X	0.97

SSVRIGSGSWK	Adcy5			
TGDFQLHTNVNDGTEFGGSIYQK	Specc1l			
TSSISALR	Tgm7		X	0.78
VHAELADVLTEVVVDSVLAVR	1110038B12Rik		X	0.53
VMPILLDSK	Phkb		X	0.61
VVLLGLSSIPSLVGHR	Fam107b	x	X	0.42
WTSEFEASLINR	chr14:57578727-57578763		X	0.81
YANNNSKY	Depdc5			
YVALDFEQEMAMAASSSSLEK	chr2:130463229-130463292			

Table S4: Human parallels of mouse non-canonical peptides. Human versions of murine non-canonical peptides found in TCGA datasets with PepQuery. Green-highlighted peptides show a decreased abundance in inflamed proximal colon samples while red-highlighted samples show an increased abundance in inflamed proximal colon samples.

Peptide	Lifted Human Coordinates	TCGA Colon Cancer	TCGA Breast Cancer	TCGA Breast Cancer PP	TCGA Ovarian	TCGA Ovarian PP	TCGA Ovarian GP	VU CC	PNNL CC	PNNL CC PP
**AAAAAAAAAAAAAASHSVAK**	chr4:72053029-72053086			x						
**ARPVSSAASVYAGAGGSGSR**	chr14:33082441-33082494		x	x	x			x		
**ASLQVSTLRLCR**	chr14:21559259-21559295		x		x					
**ELKEVIQR**	chr3:49159774-49159798		x	x	x	x				
**FSMVVQDGIVK**	chr17:13665162-13665195		x		x			x		
**LGTGAMLPLEAVK**	chr1:903676-903724	x			x					x
**LLGIDLGGK**	chrX:53654138-53654205		x	x				x		
**SKPAITGPK**	chr16:86531751-86542270	x	x	x	x	x	x	x	x	x
**SKPCISGLMVPEK**	chr1:182357758-182357797	x	x	x					x	
**VHAELADVLTEVVVDSVLAVR**	chr6:31802600-31803093	x	x	x	x	x	x	x	x	x
**VVLLGLSSIPSLVGHR**	chr10:14562824-14562872		x	x	x	x	x		x	

Figure S1: Non-canonical protein with differential abundance in global proteomics. The protein chr8: 73261429-73261687+ in the sectioned proteogenomic FASTA database was shown to be enriched in inflamed proximal colon samples. (a) Genomic coordinates associated with chr8: 73261429-73261687+, visualized with the UCSC Genome Browser. (b) Peptides associated with chr8: 73261429-73261687+ detected in Proteome Discoverer.

(a)

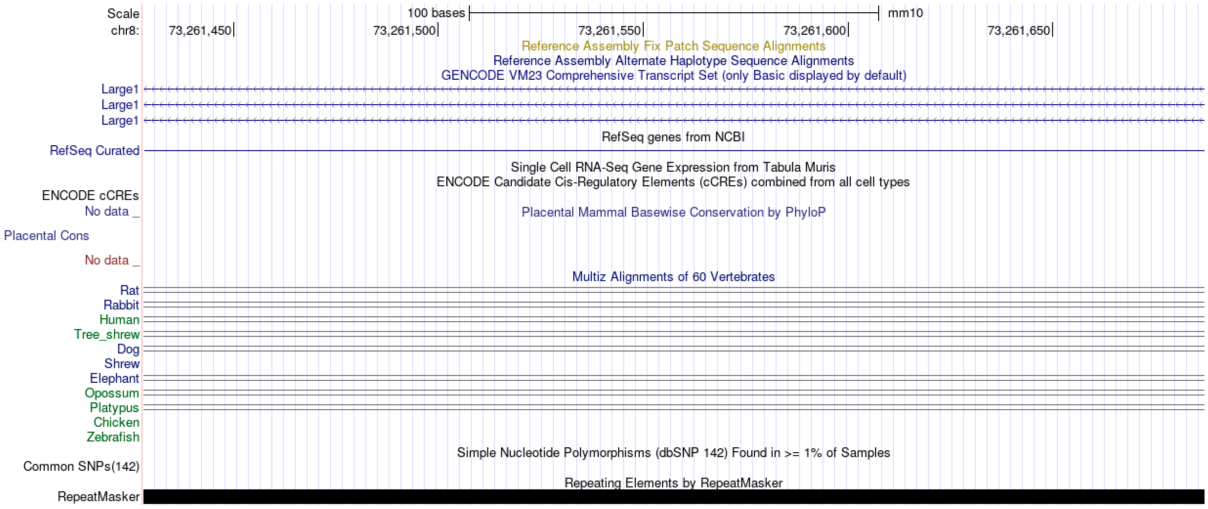

(b)

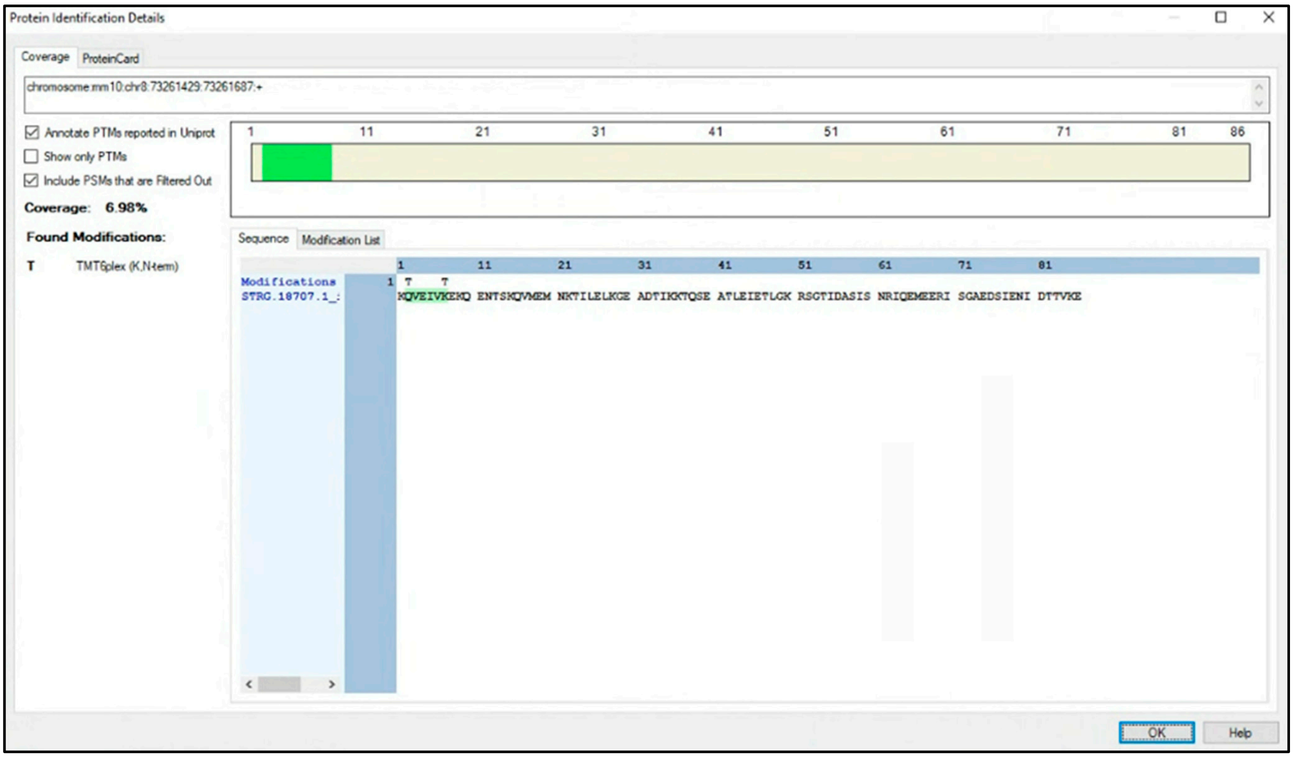

Figure S2: MS/MS spectra of example non-canonical peptides. Spectra of selected highly confident (p-value < 0.001) non-canonical peptides which passed PepQuery are presented here. Product ion deviations are expressed in ppm. Spectra were visualized using the Proteomics Data Viewer (PDV).

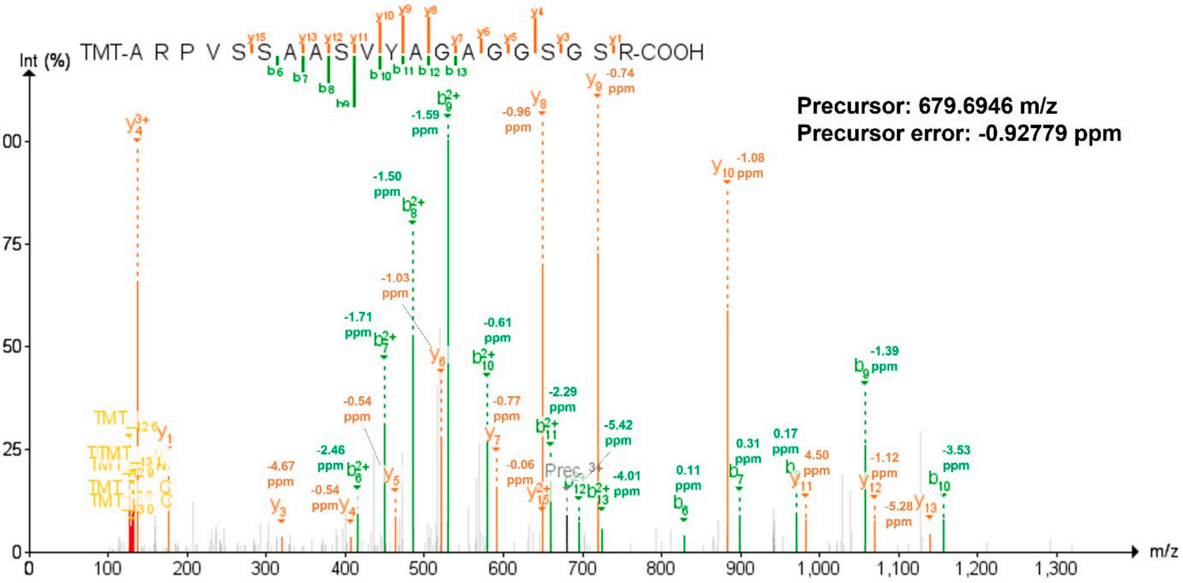

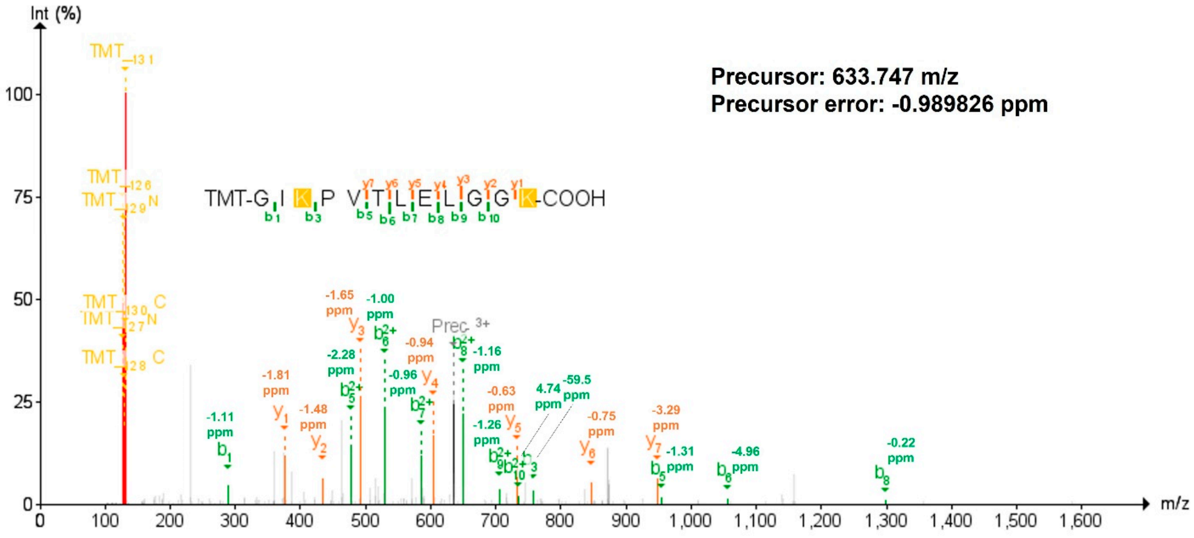

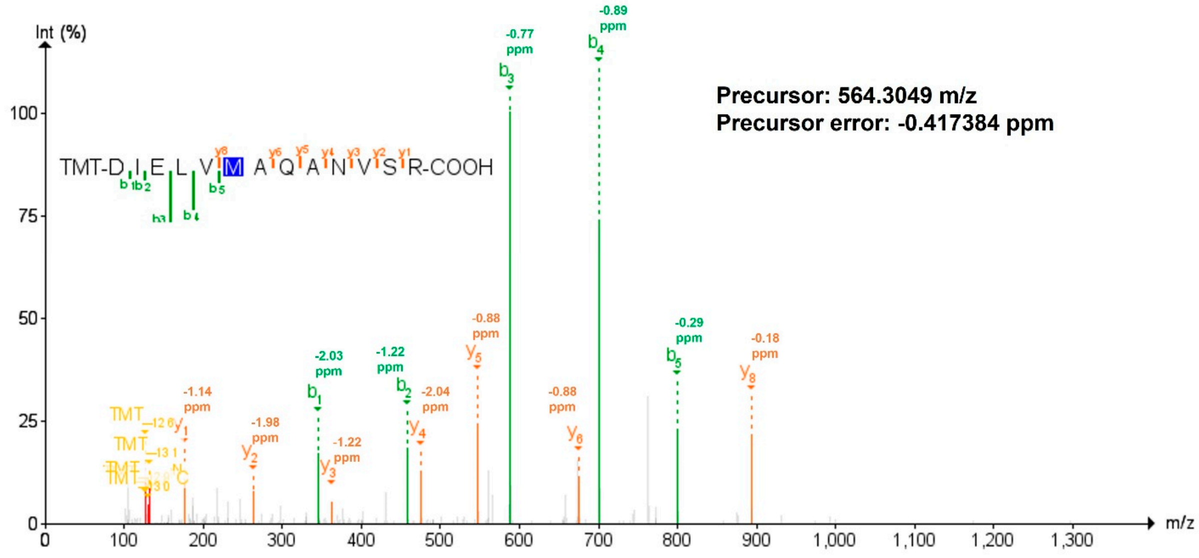
Int (%)
TMT-DIELVMAQANVSR-COOH
Precursor: 564.3049 m/z
Precursor error: -0.417384 ppm
m/z

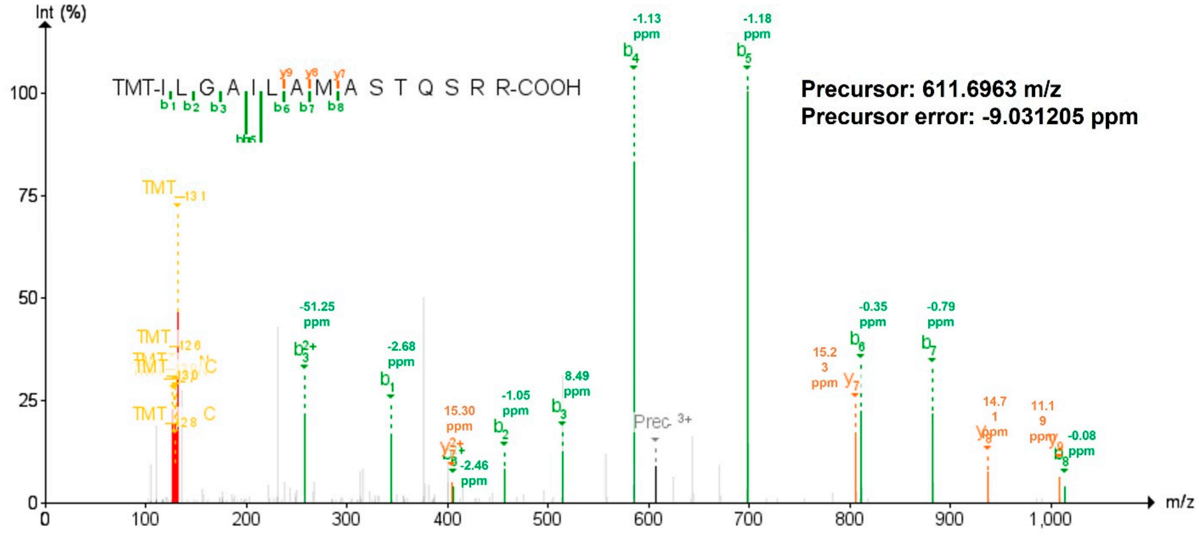
Int (%)
TMT-ILGAILAMASTQSRR-COOH
Precursor: 611.6963 m/z
Precursor error: -9.031205 ppm
m/z

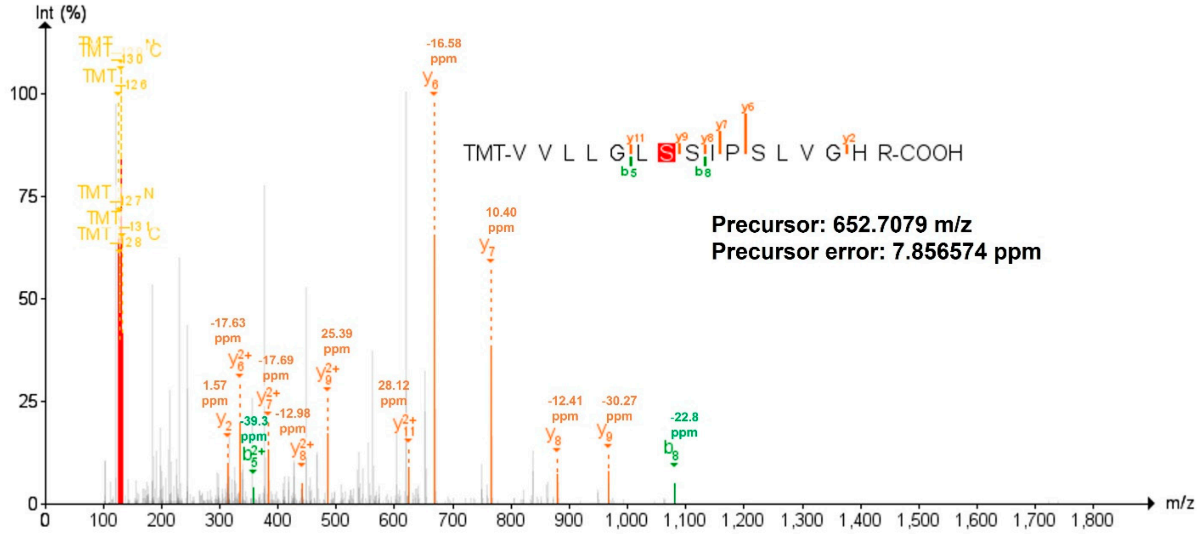

TMT-V V L L G L S S I P S L V G H R-COOH
Precursor: 652.7079 m/z
Precursor error: 7.856574 ppm
Int (%)
m/z

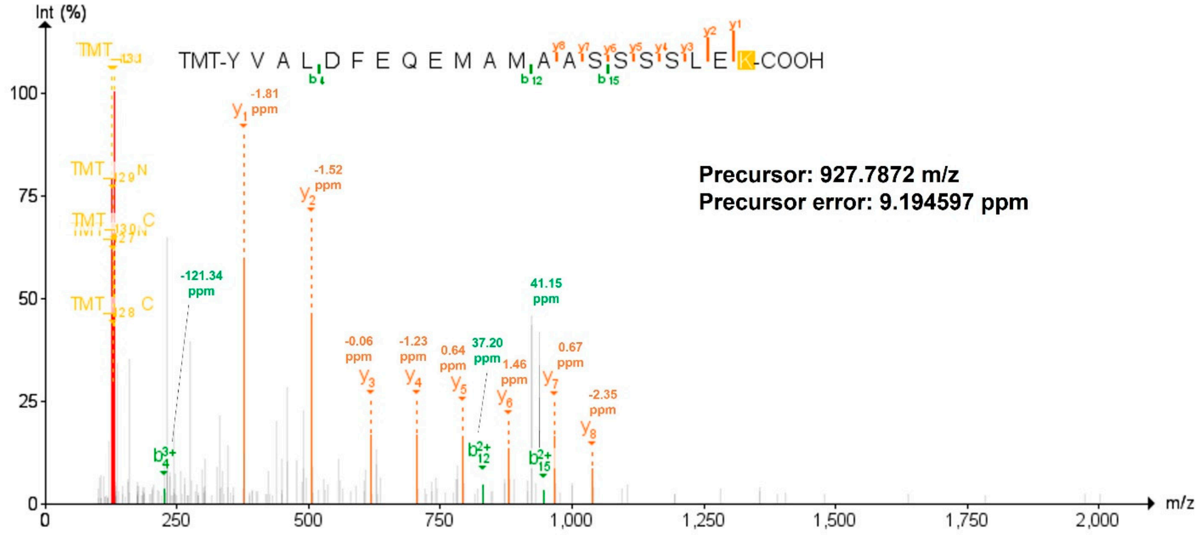

TMT-Y V A L D F E Q E M A M A A S S S S L E K-COOH
Precursor: 927.7872 m/z
Precursor error: 9.194597 ppm
Int (%)
m/z

Figure S3: Comparison of PSMs passing the Galaxy-P workflow with PSMs from MSFragger, MaxQuant. The raw proximal colon mass spectrometry data was searched against the custom FASTA database using MSFragger and MaxQuant and compared to the non-canonical peptides that were detected in and passed the Galaxy-P workflow. In both tools, STY phosphorylation, M oxidation, and N-terminus/K TMT6plex were utilized as variable modifications, Carbimidomethylation of C was used as a fixed modification. MSFragger was run in Fragpipe along with Philospher, , Percolator and ProteinProphet were used to validate the spectra, and TMT-Integrater used to quantitate the TMT6 channels.

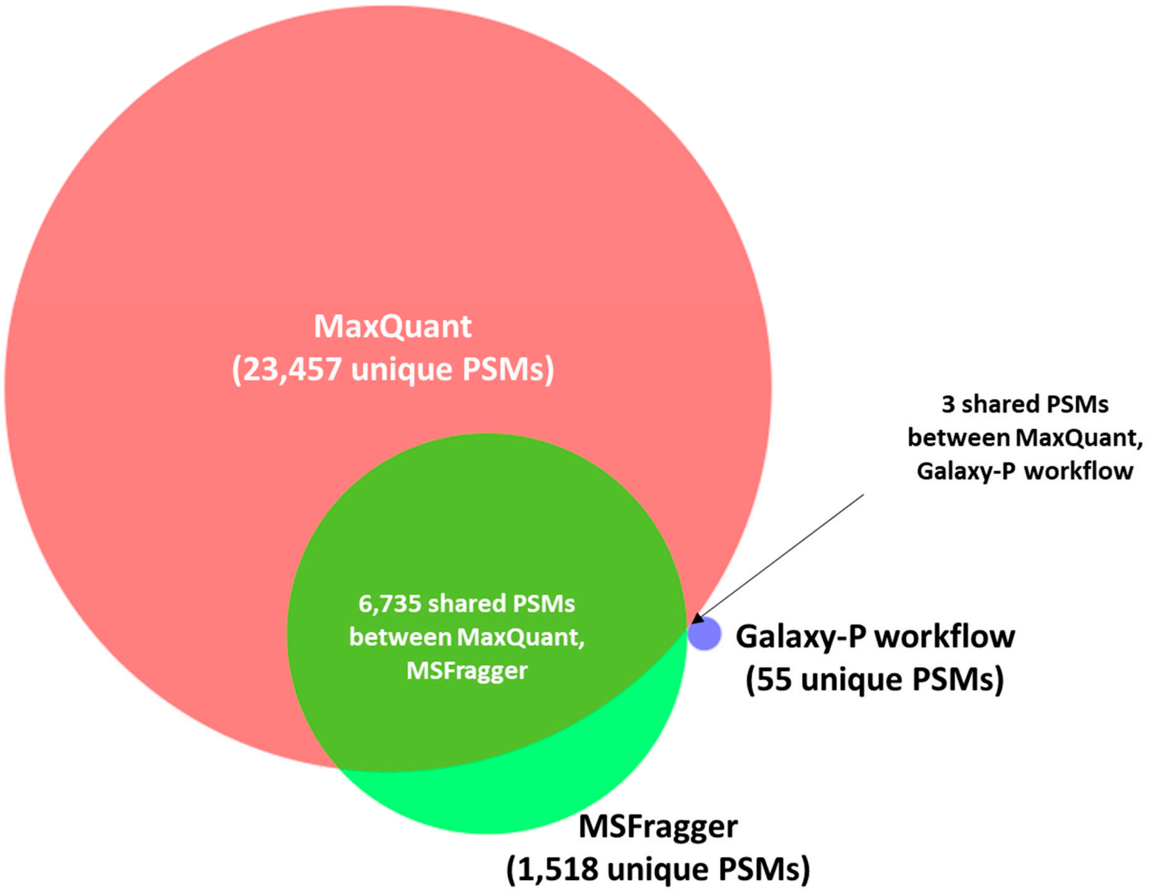

Figure S4: Genomic coordinates of non-canonical peptide AASSANIPK. The genomic coordinates of the peptide AASSANIPK, found in the 3′ untranslated region of the Sorl1 gene. This peptide was found to have a slightly increased abundance in inflamed proximal colon tissue. Genomic coordinates determined via UCSC Genome Browser.

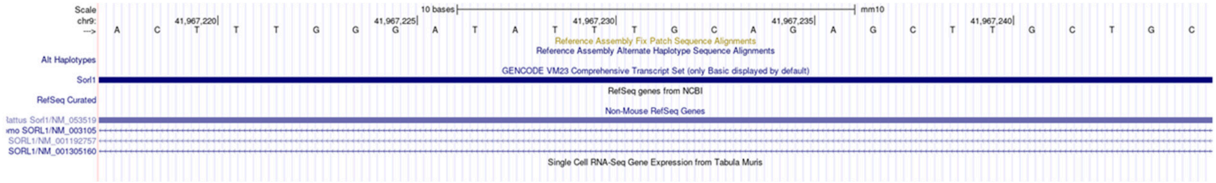

Supplement: Supplementary file 1 [file proteomes-10-00011-s001.zip › proteomes-1596773-supplementary.pdf]
